# Revealing novel biomarkers for diagnosing chronic kidney disease in pediatric patients

**DOI:** 10.1038/s41598-024-62518-w

**Published:** 2024-05-21

**Authors:** Sandra Benito, Nora Unceta, Mateusz Maciejczyk, Alicia Sánchez-Ortega, Katarzyna Taranta-Janusz, Julita Szulimowska, Anna Zalewska, Fernando Andrade, Alberto Gómez-Caballero, Pawel Dubiela, Ramón J. Barrio

**Affiliations:** 1 Department of Analytical Chemistry, Faculty of Pharmacy, University of the Basque Country (UPV/EHU), Paseo de La Universidad 7, 01006 Vitoria-Gasteiz, Spain; 2 i+Med, S.Coop Parque Tecnológico de Alava, Albert Einstein 15, 01510 Vitoria-Gasteiz, Álava Spain; 3 Department of Hygiene, Medical University of Bialystok, 15-233, Białystok, Poland; 4 Central Service of Analysis (Sgiker), University of the Basque Country (UPV/EHU), Laskaray Ikergunea, Miguel de Unamuno 3, 01006 Vitoria-Gasteiz, Spain; 5 Department of Pediatrics and Nephrology, Medical University of Białystok, Białystok, Poland; 6 Department of Pedodontics, Medical University of Bialystok, 15-274, Białystok, Poland; 7 Department of Conservative Dentistry, Medical University of Bialystok, 15-274, Białystok, Poland; 8 Metabolomics and Proteomics Platform, Biobizkaia Health Research Institute, 48903 Barakaldo, Bizkaia Spain; 9 Department of Regenerative Medicine and Immune Regulation, Medical University of Bialystok, 15-269, Białystok, Poland

**Keywords:** Biochemistry, Biomarkers, Molecular medicine, Nephrology

## Abstract

Pediatric chronic kidney disease (CKD) is a clinical condition characterized by progressive renal function deterioration. CKD diagnosis is based on glomerular filtration rate, but its reliability is limited, especially at the early stages. New potential biomarkers (citrulline (CIT), symmetric dimethylarginine (SDMA), S-adenosylmethionine (SAM), n-butyrylcarnitine (nC4), cis-4-decenoylcarnitine, sphingosine-1-phosphate and bilirubin) in addition to creatinine (CNN) have been proposed for early diagnosis. To verify the clinical value of these biomarkers we performed a comprehensive targeted metabolomics study on a representative cohort of CKD and healthy pediatric patients. Sixty-seven children with CKD and forty-five healthy children have been enrolled in the study. Targeted metabolomics based on liquid chromatography-triple quadrupole mass spectrometry has been used for serum and plasma samples analysis. Univariate data analysis showed statistically significant differences (*p* < 0.05) in the concentration of CNN, CIT, SDMA, and nC4 among healthy and CKD pediatric patients. The predictive ability of the proposed biomarkers was also confirmed through specificity and sensitivity expressed in Receiver Operating Characteristic curves (AUC = 0.909). In the group of early CKD pediatric patients, AUC of 0.831 was obtained, improving the diagnostic reliability of CNN alone. Moreover, the models built on combined CIT, nC4, SDMA, and CNN allowed to distinguish CKD patients from healthy control regardless of blood matrix type (serum or plasma). Our data demonstrate potential biomarkers in the diagnosis of early CKD stages.

## Introduction

Chronic kidney disease (CKD) represents a spectrum of disorders affecting both the structural integrity and functional competence of the kidneys. In the pediatric population, CKD poses a significant risk of severe growth retardation, metabolic derangements, cardiovascular complications, and an elevated susceptibility to neurocognitive impairment. The ultimate stage of CKD, kidney failure, often necessitates advanced interventions such as dialysis or transplantation. Early and accurate diagnosis of CKD in children is imperative to curtail disease progression and mitigate adverse outcomes^1,2^.

The etiopathology of CKD exhibits notable variations between pediatric and adult cohorts. While hypertension and type 2 diabetes mellitus are predominant causes in adults, congenital anomalies of kidneys and urinary tract, alongside hereditary nephropathies, prevail in the pediatric demographic^3,4^.

In the realm of clinical practice, kidney damage is meticulously staged based on glomerular filtration rate (GFR). GFR, a critical parameter, is commonly derived from serum creatinine concentration—a biomarker influenced by factors such as renal immaturity, age, gender, diet, weight, and muscle mass^5^. However, serum creatinine's applicability is confined, particularly in early CKD stages, as its levels may remain unaltered until substantial nephron damage occurs^6^.

Metabolomics, an evolving research methodology, emerges as a promising avenue for identifying novel prognostic and diagnostic biomarkers, thereby contributing to the identification of molecular targets for enhanced therapeutic interventions^7^. While urine analysis is often considered non-invasive, the choice between plasma and serum for diagnosis raises intriguing questions about the comparability of outcomes^8–11^.

Several metabolic studies have been performed to identify new biomarkers that could assess renal function and contribute to earlier diagnosis of CKD. Metabolomics CKD studies in blood have mainly focused on adults^12–15^ and to a limited extent, on pediatric CKD patients^16–19^.

The primary objective of this study is to explore and validate the diagnostic utility of seven potential biomarkers—citrulline (CIT), symmetric dimethylarginine (SDMA), S-adenosylmethionine (SAM), n-butyrylcarnitine (nC4), cis-4-decenoylcarnitine (CIS4DEC), sphingosine-1-phosphate (S1P), and bilirubin (BIL)—in addition to the standard creatinine (CNN). These biomarkers have been proposed in recent literature as potential indicators for early-stage CKD diagnosis in pediatric patients^20^. Through a targeted metabolomics study, we aim to comprehensively analyze plasma and serum samples from a representative cohort of pediatric CKD patients and healthy volunteers.

## Results

### Patient characteristics

Pediatric patients suffering from CKD were classified according to estimated glomerular filtration rate (eGFR): CKD2 (60–89 mL/min/1.73m^2^), CKD3 (30–59 mL/min/1.73m^2^), CKD4 (15–29 mL/min/1.73m^2^) and CKD5 (< 15 mL/min/1.73m^2^). The clinical characteristics of the patients involved in this study are summarized in Table 1.

**Table 1:** Clinical characteristics of the patients involved in the study.

	CONTROL	CKD2	CKD3	CKD4	CKD5
Sex (M/F)	27/18	14/9	12/6	4/6	9/7
Age (2-12y/13-18y)	37/8	13/10	8/10	7/3	9/7
Treatment Not treated/Dialyzed/Transplanted	45/0/0	17/0/6	16/0/2	10/0/0	2/13/1
Blood sample type Plasma/Serum	13/32	15/8	6/12	5/5	5/11

### Evaluation of biomarkers diagnostic value

Seven new potential biomarkers (CIT, SDMA, SAM, nC4, CIS4DEC, S1P, and BIL) and current standard, CNN, were analyzed in blood-derived samples by a validated liquid chromatography-triple quadrupole mass spectrometry (LC-QQQ-MS) methodology. Concentrations of CIT, CNN, SDMA, SAM, nC4, CIS4DEC, S1P, and BIL are summarized in Table 2. Descriptive statistics are shown as the median and 3rd-97th interquartile range. CIT, SDMA, nC4, CIS4DEC, and S1P are up-regulated in CKD patients’ blood samples, while SAM and BIL seem to be down-regulated comparing to the healthy volunteers.

**Table 2:** Results obtained for the proposed biomarkers expressed as median value in mg/L (3rd-97th interquartile range).

Compound	Control	CKD
CIT	4.13 (1.52–32.65)	8.14 (1.48–59.11)*
CNN	4.55 (1.99–10.98)	16.85 (3.04–77.43)*
SDMA	0.12 (0.06–0.27)	0.27 (0.07–0.86)*
nC4	0.015 (0.003–0.046)	0.034 (0.004–0.198)*
SAM	0.18 (0.01–0.95)	0.15 (0.02–1.44)
CIS4DEC	0.32 (0.03–6.61)	0.43 (0.07–6.03)
S1P	0.51 (0.17–1.43)	0.60 (0.27–1.82)
BIL	7.07 (0.21–270.42)	5.38 (0.02–201.85)

*p* value is expressed as *p < 0.05.

Notably, CNN, CIT, SDMA, and nC4 demonstrated significant differences between CKD patients and healthy volunteers (*p* < 0.05, Table 2).

### Multivariate analysis

**Figure 1:**
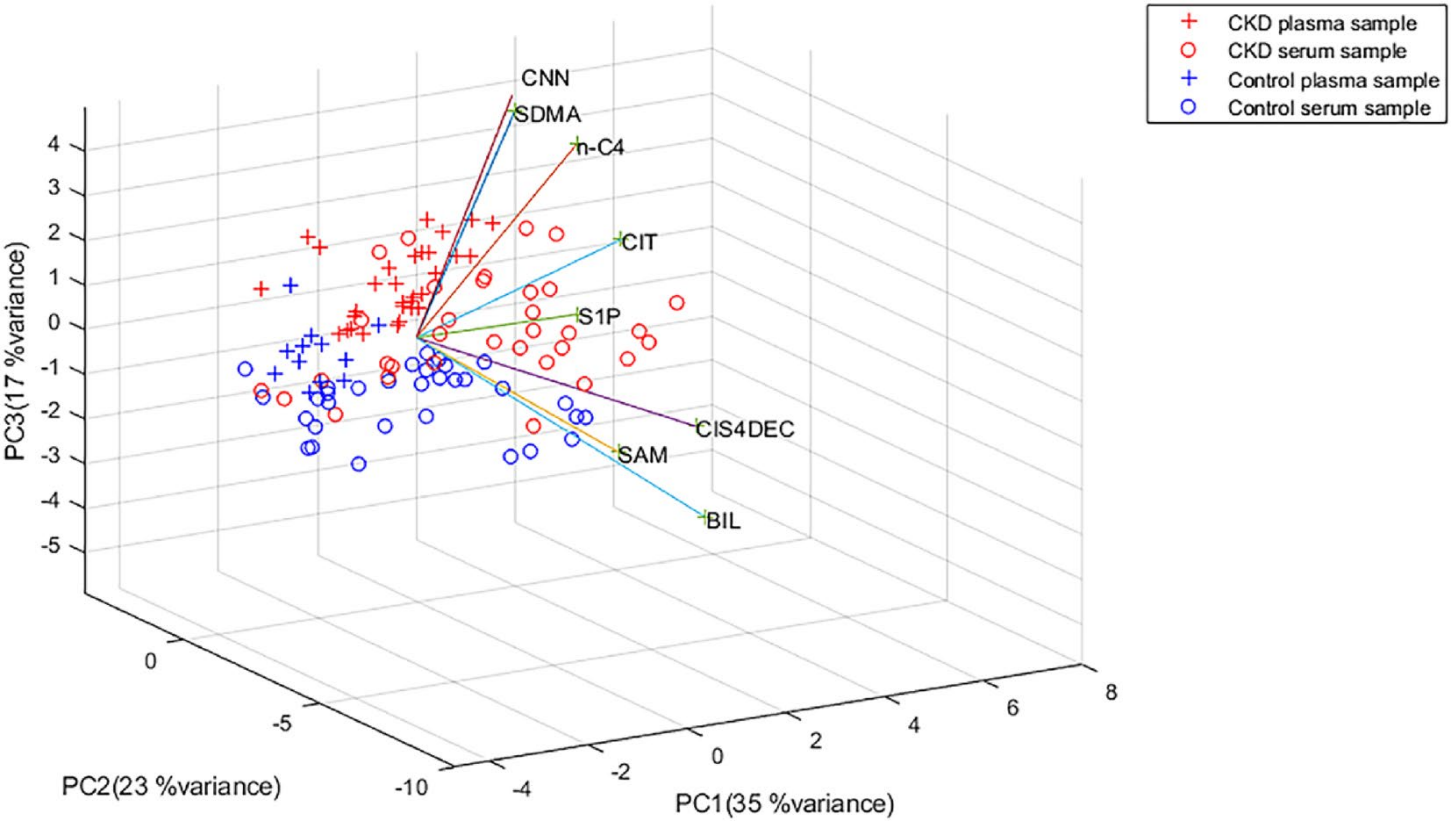
PCA biplot of CKD and control patients for all the metabolites colored by blood sample type.

**Figure 2:**
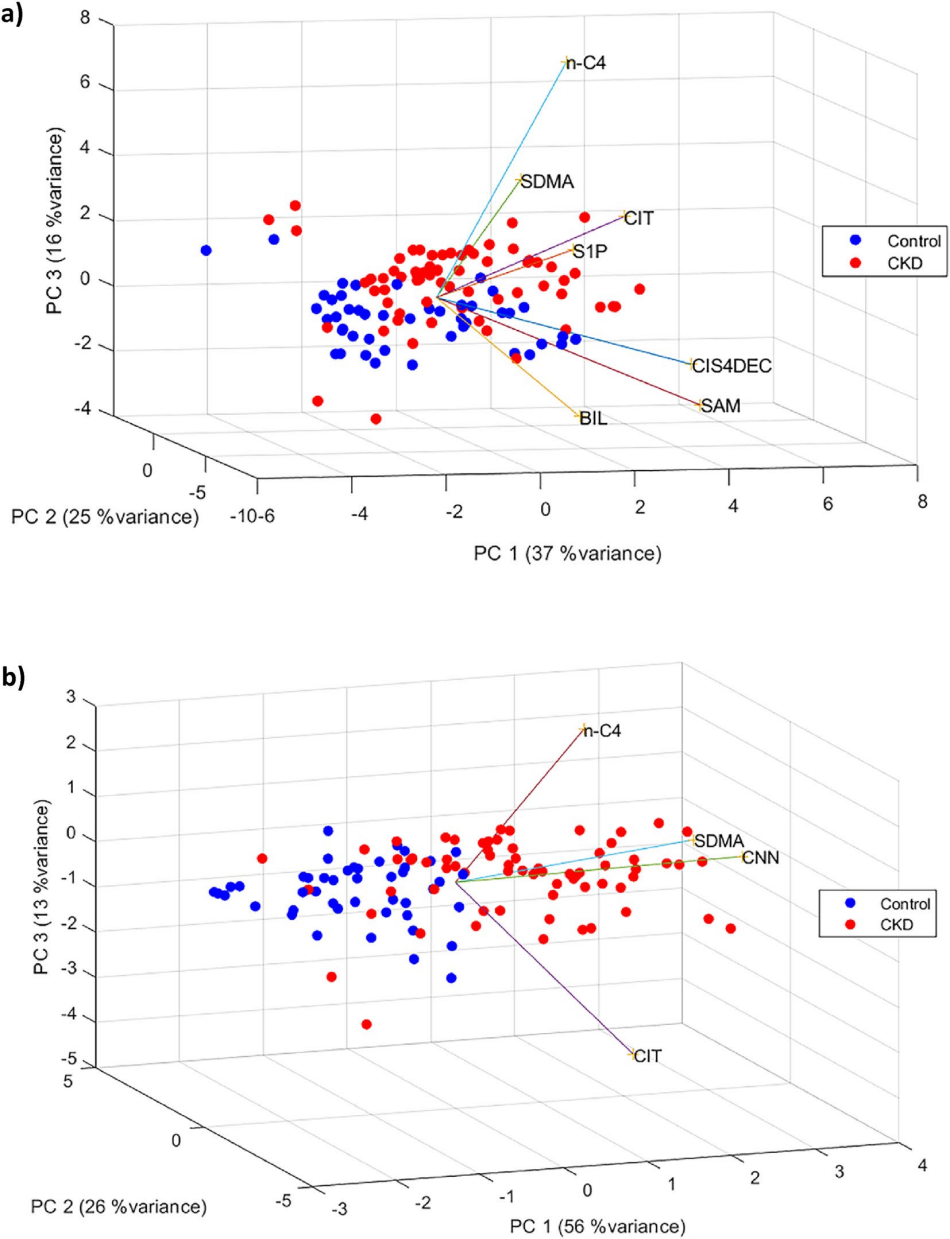
PCA biplot for control and CKD patients for all the analytes except for CNN (**a**) and CNN, CIT, SDMA, and nC4 (**b**).

Principal component analysis (PCA) revealed clear separation between the control group and CKD patients based on the concentrations of the eight metabolites, accounting for a variance of 75% (Fig. 1). A separate PCA excluding CNN demonstrated similar efficiency in group differentiation (variance of 78%, Fig. 2). Focusing on CNN, CIT, SDMA, and nC4 increased the variance to 91% in plasma and 93% in serum samples (Fig. 2b).

Partial Least-Squares Discriminant Analysis (PLS-DA) resulted in a discrimination accuracy of 81% using eight biomarkers and 80% using CNN, CIT, SDMA, and nC4 (data not shown).

The cross-correlations between the seven novel analytes to CNN were evaluated. The positive outcome was found only between SDMA and CNN (r = 0.6812, *p* < 0.001, data not shown).

**Figure 3:**
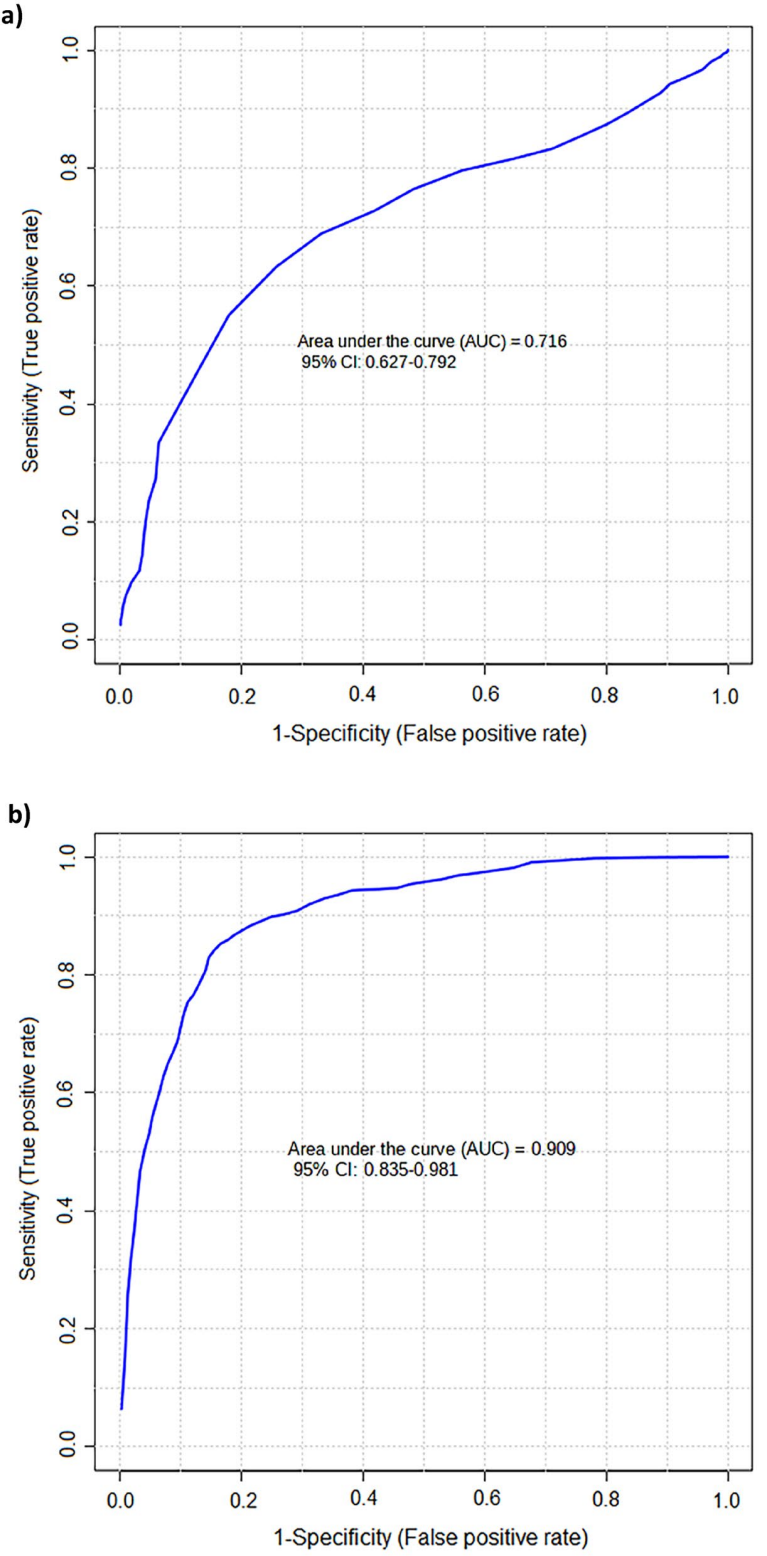
ROC multivariate curve built using all the analytes (**a**) and CNN, CIT, SDMA, and nC4 (**b**) in CKD and healthy control patients regardless of blood sample type.

A receiver operating characteristic (ROC) curve analysis demonstrated area under the curve (AUC) of 0.671 for CNN alone, while multivariate analysis including eight metabolites improved AUC to 0.716. The combination of CNN, CIT, SDMA, and nC4 provided AUC of 0.909, underscoring their potential for discriminating between control and CKD patients (Fig. 3).

### Clinical value of biomarkers in detection of early-stage CKD patients

Samples from patients classified into CKD stage 2 were subjected to PCA model. Likewise, diagnostic discrimination was improved between control patients and early CKD patients when PCA model was built with CNN, CIT, SDMA, and nC4 and reached variance of 89%. Analysis of eight metabolites resulted in variance of 72%.

PLS-DA analysis was carried out to differentiate between healthy and early stages CKD pediatric patients. Accuracy of 78% was obtained with all the analytes, while testing only CNN, CIT, SDMA, and nC4 resulted in similar results (77%).

Multivariate ROC curves including eight analytes reached AUC = 0.856. Analysis of four metabolites, CNN, CIT, SDMA, and nC4 resulted in AUC = 0.831 while CNN alone demonstrated AUC of 0.737 (data not shown).

### Impact of age, sex, and treatment on the biomarkers level

Sex did not impact statistical differences in CNN, SDMA, nC4, and CIT according to the Mann–Whitney U test (*p* > 0.05) and the difference was unsignificant also in the subgroup analysis of adolescents (13–18 years old; *p* > 0.05).

Impact of age on CNN, CIT, SDMA, and nC4 concentrations, was evaluated in two groups: ≤ 12-year-old children and > 12-year- old adolescents. No age-related difference was observed for SDMA, nC4, and CIT (*p* > 0.05). The only biomarker differing significantly increased in adolescent group was CNN (*p* < 0.001, Suppl. Table 1 and Suppl Table 2.).

**Figure 4:**
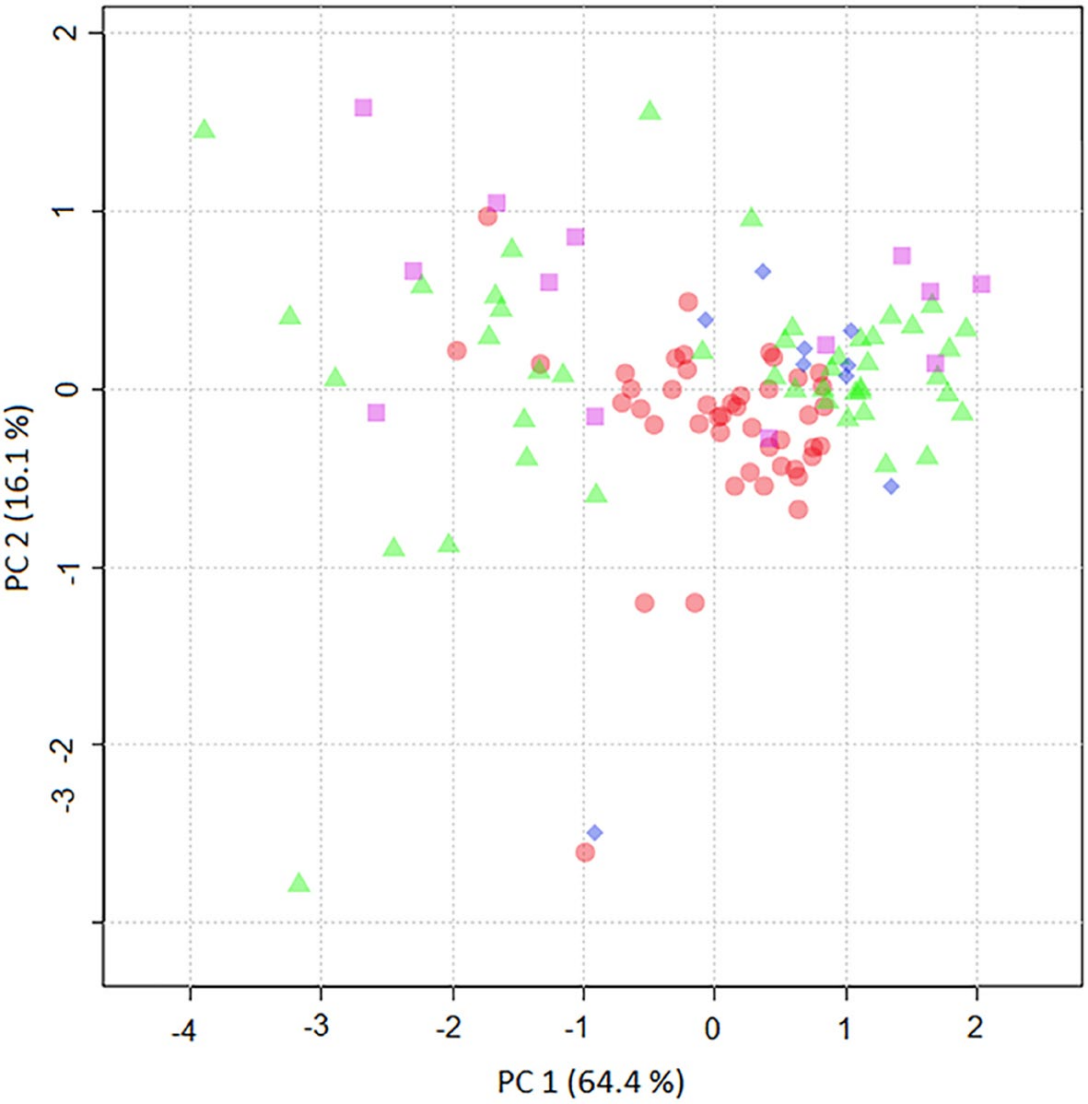
PCA score plot built using CNN, SDMA, nC4, and CIT in control (red circle), untreated (green triangle), transplanted (blue rhombus)), and dialyzed (purple square) CKD patients.

Finally, the effect of the treatment on the concentration of tested metabolites was analyzed by building a PCA model. No significant differences were observed between untreated, transplanted, and dialyzed CKD. However, when control patients were also included in the PCA model, the spatial distribution of samples from transplanted patients overlapped control samples suggesting a tendency to equalize the profile of these biomarkers after receiving a transplant (Fig. 4).

## Discussion

CKD in the pediatric population poses unique challenges due to its diverse etiologies and the potential for severe long-term consequences. Early diagnosis is paramount, as timely intervention can significantly alter the trajectory of the disease, mitigating the risk of complications such as growth retardation and cardiovascular diseases^1,2^. CKD is one of the major public health problems worldwide showing pediatric prevalence of 15–74.7 cases per million children^21,22^. The real onset of CKD in children, especially in developing countries, is unknown as most of the children in early stages remain undiagnosed. Our multicenter study aimed to validate seven novel biomarkers with possible application in diagnosing early stage of CKD. The predictive ability shown in this study for CIT, SDMA, and nC4 as add on to CNN confirms that metabolite panel could be of clinical value for diagnosing early stages CKD pediatric patients.

The data clearly shows that CIT, nC4, and SDMA, in addition to CNN, in serum and plasma are up regulated in CKD pediatric patients comparing to healthy children. CIT is a derivative amino acid produced by conversions of free amino acids^23^. It is also a direct substrate for arginine biosynthesis involved in endothelial nitric oxide (NO) production. However, in CKD, activating the renin–angiotensin–aldosterone system impairs NO formation and stimulates NADPH oxidase (NOX), promoting renal failure. Reduced NO bioavailability is also a cause of CKD complications such as hypertension, obesity, and cardiovascular disease^24–26^. Not surprisingly, angiotensin II-converting enzyme inhibitors (ACEi) and angiotensin receptor 1 blockers have been used for nephroprotective treatment for years, normalizing blood pressure and reducing proteinuria. Although our study does not aim to explain this phenomenon, an increased CIT level could be potentially explained by the low arginine/glycine amidinotransferase activities^27^. Our results are in line with the previous report showing that high concentration of CIT is significantly associated with CKD incidence^28^.

The primary role of NO produced by vascular endothelial cells is vasodilation, which is possible due to the relaxation of the vascular wall smooth muscles. By lowering vascular resistance, it participates in the regulation of arterial pressure and inhibits platelet aggregation. The reduction of NO bioavailability in CKD children may be caused by arginine deficiency, increased oxidative stress, and enhanced concentration of the inhibitor of nitric oxide synthase – asymmetric dimethylarginine (ADMA)^24–26^. SDMA is the biologically inactive stereoisomer of ADMA^29^. It acts as an endogenous inhibitor of the arginine-dependent NO pathway, competing with arginine to introduce into cells^30^. Both CNN and SDMA are mainly excreted in the urine. Renal hypofunction could lead to their accumulation in blood, which explains the mutual correlation between CNN and SDMA observed in our study. Since SDMA is a biometabolite of NO, an increase in its levels also indicates vascular endothelial dysfunction in CKD children. It should be noted that SDMA is less dependent on the patient's hydration status and muscle mass than urea and creatinine, demonstrating the high diagnostic utility of this biomarker^31^. Our results confirm the outcomes of previous reports^32^ and add strong evidence to apply SDMA in clinical practice for pediatric onset of early-stage CKD patients.

nC4 is an acylcarnitine elevated in patients with short-chain acyl-CoA dehydrogenase dysfunction^33^. Increased plasma acylcarnitine has been related to adult kidney failure. It is known that the accumulation of acylcarnitine in plasma is a consequence of decreased renal clearance of the esterified carnitine fraction in patients who are not undergoing hemodialysis^34^. Our research adds to the picture of this metabolite understanding by showing its role in early stage of CKD in pediatric patients. Intra- or inter-individual factors may impact secondary metabolism-related pathways such as the arginine-creatinine metabolic pathway and arginine methylation.

Testing new biomarkers in pediatric population usually raises the question on age and sex dependent accuracy. Only CNN was age dependent as found in other studies^35^. Since other biomarkers (CIT, SDMA, and nC4) were found independent of tested factors, they seem promising CKD diagnostic candidates for both, pediatric and adult population.

The strength of the study is the analysis of both sample types, serum, and plasma. According to López-Bascón et al., critical pathways such as the citric acid cycle, metabolism of amino acids, fructose, mannose, glycerolipids metabolism, and pentose and glucuronate interconversion may be affected by the process of obtaining plasma and serum^36^. During clotting, the levels of some metabolites could be increased due to their release from blood cells or the activation or release of enzymes^37,38^. Moreover, clotting time may vary between blood samples providing higher inter-individual variability that could mask the real behavior of some metabolites^39^. The anticoagulant employed to obtain plasma could also condition amino acids, carboxylic acids, and sugar alcohol profiles^40^. Since our results were reproducible in both settings, their value is indisputable.

The study's limitation includes the presence of differences in age among the two cohorts, which may introduce a potential bias in the outcomes, as age-related physiological variations can impact metabolites level. The study is also possibly constrained by the relatively small sample size, potentially affecting the robustness and generalizability of the findings due to limited statistical power and increased susceptibility to chance variations.

Summarizing, our findings contribute valuable insights into the early diagnosis of pediatric CKD, offering a potential paradigm shift in clinical practice. The identified biomarkers, particularly when considered collectively, show promise in enhancing diagnostic precision and facilitating timely interventions. Further research is warranted to validate these findings in larger cohorts, elucidate underlying mechanisms, and explore the translational potential of these biomarkers in routine clinical settings. The integration of these biomarkers into routine clinical practice may pave the way for improved outcomes in pediatric CKD patients.

## Materials and methods

### Chemicals and reagents

N^G^,N^G´^-dimethyl-L-arginine di(p-hydroxyazobenzene-p´-sulfonate) (SDMA), S-adenosyl-L-methionine (SAM), n-butyryl-L-carnitine (nC4), iso-butyryl-L-carnitine (iC4) and citrulline (CIT) were obtained from Sigma-Aldrich (Steinheim, Germany). Creatinine (CNN) was provided by Alfa Aesar (Karlsruhe, Germany). D-erytro-sphingosine-1-phosphate (S1P) was bought from Larodan AB (Limhamn, Sweden). Cis-4-decenoylcarnitine was synthesized by Lumila research group at the Autonomous University of Madrid (Madrid, Spain). Bilirubin (BIL) was purchased from TCI (Tokyo, Japan). Isotopically labeled compounds, N^G^,N^G^´-dimethyl-L-arginine-d6 (SDMA-d6) and creatinine-d3 (CNN-d3) were supplied by Toronto Research Chemicals, TRC-Canada (North York, Canada). Acetonitrile was obtained from Scharlau (Sentmenat, Spain), LC–MS grade formic acid from FisherScientific (Ghent, Belgium), ammonium formate from Sigma-Aldrich (Steinheim, Germany) and perfluoroheptanoic acid 96% (PFHA) from Acros Organics (New Jersey, USA). The mobile phase was filtered through 0.1 μm filters from Millipore Omnipore (Watford, Ireland). Acetic acid was supplied by Fisher Scientific (Loughborough, UK).

### Study samples

Blood-derived samples were collected from sixty-seven CKD children and forty-five healthy pediatric volunteers from two countries, as described below.

We employed the Schwartz formula to define CKD in our study. The Schwartz formula utilizes serum creatinine levels, height, and an empirical constant to estimate the Glomerular Filtration Rate (GFR). This formula has been widely accepted and utilized as an enrollment criterion in various studies, including the Chronic Kidney Disease in Children study. This National Institutes of Health–funded North American cohort study aims to recruit children and adolescents with mild to moderate CKD. Its primary objectives include characterizing disease progression and assessing the impacts of CKD on cardiovascular health, growth parameters, and behavioral indices.

Plasma samples were collected at Cruces University Hospital in Barakaldo (Basque Country, Spain). Blood samples from thirty-one CKD patients, aged 3–17 (average age: 10.9 years old), and thirteen healthy control patients aged 6–16 (average age: 9.8 years old). The study protocol was approved by the Ethics Committee of Clinic Research of Cruces Hospital (approval number: E08/62). Patients and patients’ caregivers all provided informed consents. The study was performed in compliance with Spanish law on biomedical research (Law 14/2007, of July 3, on Biomedical Research, BOE no159, pp28826–28,848). Blood was drawn in the morning after overnight fasting in Beckton Dickinson EDTA tubes (Plymouth, UK). After centrifugation at 1000 g for 5 min at 4 °C, plasma samples were stored at − 80 °C until further analysis.

Serum samples were collected at the Department of Pediatrics and Nephrology of the Medical University of Bialystok (Podlasie Province, Poland). Blood samples were obtained from thirty-six CKD pediatric patients aged from 3 months to 17 years old (average age: 11.6 years) and thirty-two healthy controls aged from 1 week to 17 years old (average age: 5.9 years). The study was approved by the Institutional Review Board of the Medical University of Bialystok (R-I-002/301/2019). Blood was drawn in the morning after overnight fasting in Beckton Dickinson serum tubes (Plymouth, UK). Blood samples were left to clot and then centrifuged at 1000 g for 5 min at 4 °C. Serum samples were stored at − 80 °C until further analysis.

The exclusion criterium in both control and study groups was the occurrence of systemic (e.g., metabolic, autoimmune, neoplastic, and infectious) diseases. Children taking non-steroidal anti-inflammatory drugs (NSAIDs), glucocorticosteroids, hormones, antibiotics, vitamins, and dietary supplements for at least three months before the study were also excluded.

### Sample processing

After thawing at room temperature, 50 µL of a sample was placed in Eppendorf tubes and spiked with 10 µL of a mix solution containing 100 µg mL^−1^ of creatinine-d3 and 1 µg mL^−1^ of SDMA-d6. Then, protein precipitation was performed by adding 150 µL of cold acetonitrile, vortexing the mixture, and centrifuging for 10 min at 15,600 g at 4 °C. The obtained supernatant was transferred to chromatographic vials and evaporated in a nitrogen stream in a Techne, Dri-Block®DB-3D (Staffordshire, UK) evaporator system. Finally, the residue was reconstituted in 100 µL of acetonitrile: acetic acid 0.5 M (75:25; v:v) solution and 2 µl were analyzed using LC-QQQ-MS.

### LC-QQQ-MS method

Chromatographic analysis of blood samples was performed on an Agilent 1200 Series HPLC system coupled to Agilent 6410 Series triple quadrupole mass spectrometer (LC-QQQ-MS) from Agilent Technologies (Santa Clara, CA, USA), equipped with an electrospray source (ESI). Chromatographic separation was carried out in a Zorbax Eclipse Plus C18 (3.0 × 50 mm, 1.8 µm) reversed-phase column preceded by a C8 guard column (2.1 × 12 mm, 5 µm), both from Agilent Technologies. The mobile phase composition, gradient program, and column temperature were published previously^20^. Mass spectrometry measurements were recorded in the following conditions: fragmentor voltage, 100 V; drying gas temperature and flow, 250 °C and 10 L/min; nebulizer pressure, 40 psig; and capillary voltage, 3500 V. Quantification was performed by dynamic multiple reaction monitoring (dMRM) mode. Retention time, 1 precursor ion, and 2 product ions were used to identify and quantify the analytes in the samples^20^. iC4 and its structural isomer nC4 present the same ionization pattern in MS and MS/MS modes, thus it was necessary to include both isomers in the method to ensure the correct identification and quantification of nC4. This analytical method was previously evaluated in terms of linearity, trueness, precision, limits of quantification, and stability, as described elsewhere^20^.

Agilent Mass Hunter Workstation Data Acquisition version B.08.00 software and Mass Hunter Qualitative Analysis version B.07.00, both Agilent Technologies, were used for data acquisition and raw data processing. Data analysis was carried out using SPSS Statistics 23 from IBM (Armonk, New York, USA), Metaboanalyst, and Matlab R2015a (Mathworks, Natick, Massachusetts, United States).

### Data analysis

Univariate data analysis was carried out to find whether differences in biomarkers concentration between CKD and control groups were significant. The distribution of blood concentration of each analyte in control and CKD patients was evaluated with the Kolmogorov–Smirnov test. The student's t-test was applied for parametric variables, whereas the Mann–Whitney U test was applied to non-parametric variables.

The intrinsic interdependency of the metabolite concentrations was evaluated by multivariate data analysis. Logarithm transformation of data was performed to analyze the data set in a normal distribution range. Subsequently, due to different concentration ranges of metabolites, data was Pareto scaled by normalizing the concentration of each analyte, subtracting the mean metabolite concentration, and dividing it by the square root of its standard deviation. Then, a PCA model was built for all samples.

The predictive ability of the proposed biomarkers was analyzed by ROC curves that describe the relationships between the sensitivity and specificity. Classical univariate ROC curve analysis was performed for CNN alone. In addition, multivariate ROC curves were generated using the PLS-DA algorithms for building the classification method using two-thirds of the samples and validating the approach on the 1/3 samples left out.

All data (concentration of the analytes) were log transformed before data normalization by Pareto scaling.

### Institutional review board

The protocol of the study was approved by the local Ethical Committee of Clinic Research of Cruces Hospital (approval number: E08/62) and the Medical University of Bialystok, Poland (number R-I-002/301/2019).

### Supplementary Information

Supplementary Information.

## Data Availability

The data that support the findings of this study are not openly available due to reasons of sensitivity and are available from the corresponding author upon reasonable request. Data are located in controlled access data storage at the institutions participating in the study.
