# Supplementary material for: Revealing novel biomarkers for diagnosing chronic kidney disease in pediatric patients
Source: Sci Rep. 2024 May 21;14:11549. doi: 10.1038/s41598-024-62518-w (PMC11109104; doi:10.1038/s41598-024-62518-w)
Supplement: Supplementary file 1 — Supplementary Information. [file 41598_2024_62518_MOESM1_ESM.docx]

**Suppl. Table 1**

Levels of selected biomarkers expressed as median value in mg/L for ≤12-year-old children (3rd-97th interquartile range).

| **Compound** | **Control (n=41)** | **CKD (n=38)** |
| --- | --- | --- |
| CIT | 4.13 (1.50-34.98) | 8.87 (1.73-71.09)* |
| CNN | 4.00 (1.86-9.50) | 15.23 (2.56-80.687)* |
| SDMA | 0.114 (0.058-0.274) | 0.266 (0.061-0.915)* |
| nC4 | 0.015 (0.004-0.047) | 0.035 (0.010-0.269)* |

*p* value is expressed as *p<0.05

**Suppl. Table 2**

Levels of selected biomarkers expressed as median value in mg/L for >12-year-old children (3rd-97th interquartile range^1^).

| **Compound** | **Control (n=8)^1^** | **CKD (n=32)** |
| --- | --- | --- |
| CIT | 4.53 (2.98-11.32) | 7.89 (1.43-31.32) |
| CNN | 9.34 (6.82-10.06) | 19.45 (3.00-58.46)* |
| SDMA | 0.128 (0.084-0.142) | 0.27 (0.07-0.700)* |
| nC4 | 0.017 (0.004-0.023) | 0.031 (0.004-0.100)* |

*p* value is expressed as *p<0.05

^1^These values correspond to 25th and 75th percentile (SPSS software does not provide 97^th^ percentile for n=8)
